# Longitudinal Randomized Cohort Study of SARS-CoV-2 Antibody Seroprevalence in the St. Petersburg Population

**DOI:** 10.3390/v14050913

**Published:** 2022-04-27

**Authors:** Vyacheslav S. Smirnov, Liudmila V. Lyalina, Anzhelika M. Milichkina, Irina V. Khamitova, Elena V. Zueva, Valery A. Ivanov, Vitaly S. Zaguzov, Areg A. Totolian

**Affiliations:** Saint Petersburg Pasteur Institute, 197101 St. Petersburg, Russia; lyalina@pasteurorg.ru (L.V.L.); amilichkina@yandex.ru (A.M.M.); ckdl@pasteurorg.ru (I.V.K.); elenazueva9@gmail.com (E.V.Z.); korsaring@yandex.ru (V.A.I.); zaguzov.vs@gmail.com (V.S.Z.); totolian@spbraaci.ru (A.A.T.)

**Keywords:** coronavirus SARS-CoV-2, seroprevalence, antibodies, nucleocapsid antigen, antigen receptor binding domain, seropositivity, St. Petersburg, volunteers, vaccination

## Abstract

**Introduction.** Since the detection of the first COVID-19 patient, 2 years have passed, during which more than 287,862,000 people have fallen ill globally, of which about 1.9% died. The implementation of SARS-CoV-2 control programs required efforts from almost all countries. An important direction in the fight against COVID-19 has been the formation of herd immunity, the main tool for managing the pandemic. **Study goal.** The aim of the study was to assess the seroprevalence of antibodies (Abs) to SARS-CoV-2 nucleocapsid (Nc) and receptor binding domain (RBD) in the St. Petersburg population during the COVID-19 pandemic. **Materials and methods.** A longitudinal cohort randomized monitoring study of Ab seroprevalence (SARS-CoV-2 Nc, RBD) was organized and conducted according to a unified methodology developed by Rospotrebnadzor with the participation of the St. Petersburg Pasteur Institute. For this purpose, a cohort was formed of 1000 volunteers who participated in all five stages of seromonitoring. The cohort was divided into seven age groups: 1–17; 18–29; 30–39; 40–49; 50–59; 60–69; 70; and older (70+) years. Seropositivity levels (Nc, RBD) were assessed by quantitative and qualitative enzyme immunoassays. During the second year of monitoring, some volunteers were vaccinated with the GamCOVIDVac (84%) or EpiVacCorona (11.6%) vaccines approved in Russia. Statistical processing was carried out using Excel 2010. Confidence intervals for shares and percentages (95% CI) were calculated using the method of A. Wald and J. Wolfowitz with adjustment (A. Agresti, B.A. Coull). The statistical significance of differences was calculated by z-test, using the appropriate online calculator (p < 0.05) unless indicated. **Results.** There was a trend toward an increase in Nc seropositivity in stages 1–3 of seromonitoring, with a decrease in stages 4–5 among children and adults. The share of RBD seropositive steadily increased during all five stages of seromonitoring. The most frequent finding was low anti-RBD Abs levels (22.6–220 BAU/mL). High Ab levels were recorded statistically significantly less frequently. Asymptomatic forms were observed in 84–88% of SARS-CoV-2 seropositive volunteers. By the fifth stage of monitoring, this indicator significantly decreased to 69.8% (95% CI: 66.1–73.4). The monitoring revealed a statistically significant increase in anti-RBD Abs alongside a statistically significant decrease in the proportion of Nc seropositives. This dynamic was especially characteristic of persons vaccinated with GamCOVIDVac. **Conclusion.** Prior to the use of specific vaccines, a seroprevalence of anti-Nc Abs was noted. After the introduction of the GamCOVIDVac vaccine in adults, a decrease in the level of anti-Nc Abs was noted due to an increase in the proportion of RBD seropositive persons.

## 1. Introduction

Two years have passed since the first case of a highly contagious infection caused by a representative of the β-coronaviruses (SARS-CoV-2) was registered. According to portal statistics [1], 287,862,113 people have been infected globally, of which 5,450,349 have died (1.89% (95% CI: 1.88–1.89) as of 31 December 2021 [1]. St. Petersburg still ranks second in Russia in terms of COVID-19 incidence. In the city, as of the end of December 2021, there were 851,908 infected, of which 28,036 people died (3.30% (95% CI: 3.25–3.33). These official data indicate a significantly higher COVID-19 mortality rate in St. Petersburg compared to the Russian average (*p* = 0.0001). St. Petersburg, over the entire period of the pandemic, showed that the infection developed in waves: periods of growth replaced by periods of decreasing case numbers. The first peak was recorded on 15 May 2020 and amounted to 541 infections per day. The second peak (29–31 December 2020) ranged from 3764–3774 infections per day. The third peak, smaller than the previous one, was marked after 7 months. A maximum of 2029 infections/day was recorded on 12 July 2021. Finally, the fourth was observed in October 2021. The daily maximum number of infected people, detected on 31 October 2021, was 3597 people [1]. It is interesting that SARS-CoV-2 vaccination, which began on 1 October 2020, did not have a visible effect on the dynamics of infection or mortality in St. Petersburg, at least until mid-December 2021. According to official data from the St. Petersburg administration, 1,983,695 people completed full vaccination as of 11 November 2021; this is about 36.8% of the total population [2].

With infections, it is known that vaccination is the determining factor of herd immunity [3,4]. According to the definition of A. Fontanet and S. Cauchemez: “herd immunity is achieved when one infected person in a population causes on average less than one secondary case” [5]. A measure of herd immunity is the basic reproductive number (Ro), which is understood as the number of individuals who will be infected in a fully susceptible population by one SARS-CoV-2 carrier during one infectious period [6]. In the pre-vaccination COVID-19 period, Ro values varied widely from 1.5 to 6.5, with an average of 2.2 (95% CI: 1.4–3.9) [7,8]. Similar values are given in other works [6,9]. The indicated coefficient, substituted into the formula (Rt = 1 − 1/Ro), gives an idea of the required proportion of immune subjects in the population sufficient to stop the COVID-19 outbreak. With regard to the calculated average Ro value (3.28), this proportion was 0.695 or 69.5% [6]. Of course, SARS-CoV-2-resistant individuals in the population include not only those vaccinated but also those who have recovered from COVID-19. However, it is believed that herd immunity is not limited to specific SARS-CoV-2 immunity formed as a result of a vaccination or illness. A certain contribution is also made by measures that limit viral spread in the population. Among them, the most effective are social distancing, widespread use of personal protective gear (masks), and disinfection of environmental objects [4,5,10].

Nevertheless, despite the importance of non-pharmaceutical protective interventions, the levels of SARS-CoV-2 immunity still have a significant impact [6,11]. Despite the importance of non-pharmaceutical protective interventions, the main factor remains the level of SARS-CoV-2 immunity. In addition, analysis of the contribution of physical methods to the structure of collective immunity requires a complex mathematical approach based on metaheuristic algorithms. The results are, as a rule, probabilistic in nature [11]. Such methods provide valuable information about behavioral responses during an epidemic, but due to the complexity of mathematical analysis, they are of limited use in medical practice. 

From a practical point of view, epidemiological analysis of the state of collective immunity using serological methods (for determining the prevalence of SARS-CoV-2 Abs) and other epidemiological analysis approaches are more appropriate [12,13]. This article continues our previous publications analyzing SARS-CoV-2 Ab seroprevalence, such as our cross-sectional study of herd immunity in the St. Petersburg population [14] conducted in the early period of COVID-19 incidence (13–20 June 2020), as well as longitudinal seromonitoring of the population in 26 regions of Russia [15,16,17,18,19,20]. Features of seroprevalence in Belarus and Kyrgyzstan (2020–21) were also analyzed [21,22]. The aim of the study was to monitor the seroprevalence of Abs to Nc and RBD in the St. Petersburg population during the COVID-19 pandemic in 2020–21.

## 2. Materials and Methods

### 2.1. Formation of the Volunteer Cohort

Since June 2020, a longitudinal randomized cohort study of the dynamics of the humoral immune response to SARS-CoV-2 in the St. Petersburg population is being carried out according to a unified methodology developed by Rospotrebnadzor with the participation of the St. Petersburg Pasteur Institute [23], taking into account WHO recommendations [24]. Before initiation, the study was approved by the local ethics committee of the St. Petersburg Pasteur Institute (protocol no. 64, dated 26 May 2020). All participants, or their legal representatives, were familiarized with the purpose and methodology of the study and signed an informed consent. The study was carried out in accordance with the Declaration of Helsinki at all stages.

To participate in seromonitoring, out of 2713 volunteers examined in the primary cross-sectional study [14], 1000 people were selected, and they participated in all stages of seromonitoring without exception. The entire volunteer cohort was divided by age into 7 main groups: 1–17; 18–29; 30–39; 40–49; 50–59; 60–69; and 70^+^ years. In addition to distribution by age, the group was additionally randomized geographically in such a way that volunteer distribution (by place of residence) corresponded to the administrative territorial structure of St. Petersburg. The serological monitoring process was organized in 5 stages: stage 1 (15 June 2020–20 June 2020), stage 2 (31 August 2020–4 September 2020), stage 3 (30 November 2020–11 December 2020), stage 4 (1 March 2021–7 March 2021), and stage 5 (30 August 2021–3 September 2021).

### 2.2. Vaccination of Volunteers

Starting from 10 January 2020, marking the introduction of SARS-CoV-2 vaccines, volunteers have taken part in voluntary Russian vaccination programs. In Russia, the most commonly used vaccines are Sputnik V (GamCOVIDVac; last reg. no. LP-006395, dated 11 January 2022) and EpiVacCorona (reg. no. LP-007326, dated 2 December 2021). Both preparations were developed in Russia and are produced there [25,26,27].

In the 4th stage, a total of 14.4% of the cohort (95% CI: 12.3–16.7) were vaccinated. Of these, 91.7% (95% CI: 85.9–95.6) were vaccinated with Sputnik V (GamCOVIDVac) and 8.3% (95% CI 4.4–14.1) received the EpiVacCorona vaccine. In total, 55.5% of the cohort (95% CI: 48.3–64.6) were immunized. Among them, the shares were as follows: Sputnik V, 81.2% (95% CI: 77.5–84.5), and EpiVacCorona, 11.8% (95% CI: 9.1–14.8). In 4.1% (95% CI: 2.6–6.2), the inactivated CoviVac vaccine was used (reg. no. LP-006800, dated 19 February 2021). All the listed vaccines were used in accordance with their instructions for use. 

As follows from their published compositions [25,26], the immunity formed after Sputnik V vaccination is directed at receptor-binding domain (RBD) antigens [25], while EpiVacCorona (peptide vaccine) induces antibodies (Abs) to a complex of chemically synthesized peptide immunogens of the SARS-CoV-2 S protein [27]. CoviVac elicits an immune response to both Nc and RBD. However, the number of people receiving this vaccine was too small to have a statistically significant effect on the structure of Ab seroprevalence. The main contribution to the formation of anti-RBD Abs was made by the Sputnik V, as discussed below.

### 2.3. Analysis of the Presence of Abs to SARS-CoV-2 Nc and RBD in Volunteers

Blood plasma was used for the quantitative determination of Abs to SARS-CoV-2 antigens. For this purpose, 3 mL of blood was taken from volunteers from the cubital vein into vacutainers containing EDTA solution. Blood was centrifuged, and plasma was separated from the cellular component and used for determinations by enzyme immunoassay on a Thermo Scientific Multiskan FC device.

Assays for Nc used the “Enzyme immunoassay kit for quantitative determination of human IgG Abs to SARS-CoV-2 N protein (N-Cov-2-IgG PS), series 001” (prod. St. Petersburg Pasteur Institute, St. Petersburg, Russia). The content of anti-RBD Abs was determined by enzyme immunoassay using the “SARS-CoV-2 ELISA-IgG-screen” reagent kit (prod. LabPack, St. Petersburg, Russia). For individuals vaccinated with EpiVacCorona, Abs to its peptide components were determined by enzyme immunoassay using the “SARS-CoV-2-IgG-Vector” reagent set for detection of IgG (prod. State Scientific Center of Virology and Biotechnology “Vector”, Koltsovo, Novosibirsk region, Russia).

### 2.4. Statistical Analysis

The obtained data were processed using Excel 2010. Confidence intervals (95% CI) of shares and percentages were calculated by the method of A. Wald and J. Wolfowitz [28] with correction as per A. Agresti and B.A. Coull [29]. Correlation analysis was performed according to the Spearman method as part of the Excel 2010 package. The statistical significance of differences was calculated by z-test using a corresponding online calculator [30]. Distribution normality was tested using the Kolmogorov–Smirnov test as part of the Excel 2010 package. The statistical significance of differences, unless otherwise indicated, was assessed with a probability of *p* ≤ 0.05.

## 3. Results

### 3.1. Dynamics of SARS-CoV-2 Infection and Vaccination of the St. Petersburg Population, 2021

Previously, features of coronavirus infection and seroprevalence development dynamics (anti-SARS-CoV-2 Abs) in the Russian population in 2020 were characterized [1,15,16]. As mentioned above, the epidemic proceeded in St. Petersburg in the form of two waves (May and December 2020), of which the second was significantly higher (*p* > 0.0001). In October 2020, vaccination of the population with Sputnik V began. Initially the rate was very low, and it was not sufficient to have a significant impact on the dynamics of COVID-19 incidence.

**Figure 1:**
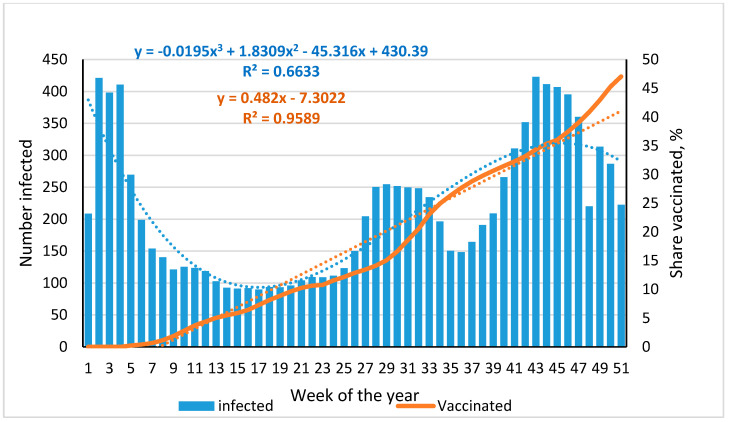
Dynamics of SARS-CoV-2 infection and vaccination of the St. Petersburg population in 2021. The blue dotted line is the infection trend; the regression equation and the coefficient of determination are highlighted in blue and placed at the top center. The brown dotted line is the vaccination trend; the regression equation and the coefficient of determination are highlighted in brown and placed at the top center. The main vaccine used in the Russian Federation was GamCOVIDVac (Sputnik V).

In the first three weeks of 2021, the decline in incidence continued after the December 2020 peak, with vaccination rates close to zero (Figure 1, Table S1). Subsequently, two parallel processes were noted: the proportion of infections decreased; and the percentage of vaccinations gradually increased. By the 12th week, however, infection dynamics stabilized and remained at the achieved level for 14 weeks, despite the fact that the proportion of those vaccinated during this period increased from 3.7% to 11.6%. The accumulated level of post-vaccination immunity was almost sixfold lower than the postulated minimum level for herd immunity [6]. 

Such a low level of post-vaccination immunity did not have a significant impact on infection dynamics. At the beginning of July 2021 (week 27), a 1.5-fold increase in incidence was recorded; it continued until August 2021 (week 33). Starting from week 39, when the proportion of fully vaccinated reached 30.5%, the incidence, despite this, began to increase rapidly. By week 43, it reached the January level (422.7‱ **0**); the proportion of those vaccinated was only 34.3%. The noted rise turned out to be short-lived. From week 44, it was already replaced by a downward trend, which continued into the first weeks of 2022. The level of vaccination coverage at the same time came close to the 50% milestone. In this regard, it is logical to assume that a further increase in post-vaccination immunity may lead to a decrease in the intensity of the COVID-19 epidemic in St. Petersburg, provided that the new, highly contagious omicron SARS-CoV-2 strain will not require any emergency measures and/or booster vaccination of already vaccinated citizens [31,32,33].

### 3.2. Seroprevalence of Anti-Nc Abs in the Volunteer Cohort, by Seromonitoring Stage

**Figure 2:**
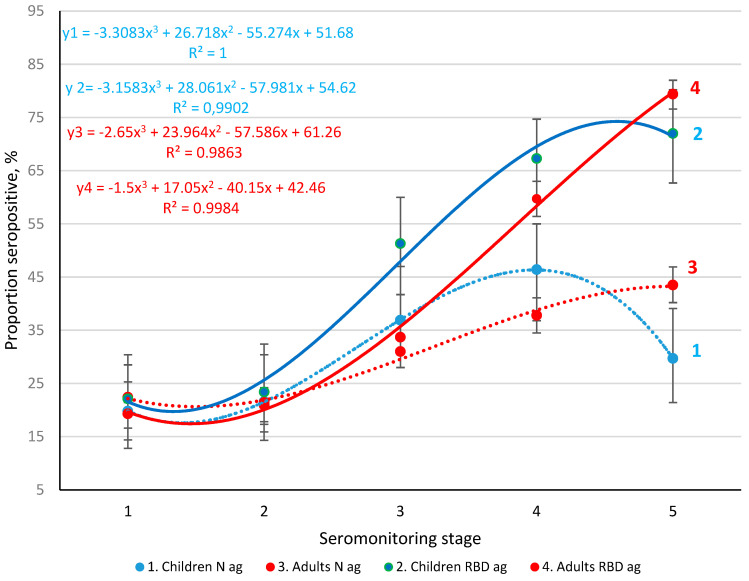
Distribution of seropositivity to N and RBD antigens (ag) in children and adults, by seromonitoring stage. Colored curves are trend lines for the percent seropositive among 1—children (N ag); 2—children (RBD ag); 3—adults (N ag); and 4—adults (RBD ag). Regression equations and coefficients of determination, in their corresponding colors, are shown in the upper left (y2, y4) and lower right (y1, y3) corners. Black vertical bars are the 95% confidence intervals. The summarized data are given in Table S1.

Seroprevalence and Ab levels (anti-Nc, anti-RBD) were determined during monitoring [34]. Taking into account the different roles of these viral antigens in the infectious process, one would expect changes in their corresponding Abs ratios in the population during the pandemic (Figure 2). The level of Nc Ab seroprevalence among children increased during stages 2–4 but decreased significantly by stage 5 (*p* < 0.001). The dynamics were described by the third-degree polynomial curve equation: y = 3.3083x^3^ + 26.719x^2^ − 55.274x + 51.68. Note that the coefficient of determination in this case was 1.0.

Seroprevalence to Nc manifested differently in adults. At stages 1–4, the shape of the trend curve was close to that of children; only seropositivity levels were lower than in children. Statistically significant changes appeared only in the fifth stage. While Ab levels decreased in children, they increased significantly in adults (Figure 2 curves 2 and 4, Table S1). The low proportion of seropositive individuals in the first stage already began to grow by the second stage. In the third stage, increases reached significant values in most volunteer age groups (Table S1). By the fifth stage, the differences reached a maximum value with tgα equal to 1.6 (in the section of the curve between stage 4 and 5). This suggests that the share of seropositive adults increased to a limit close to 100%. Naturally, such prevalence likely has a positive effect in reducing Ro and the level of overt morbidity. The above results contain only information about the seroprevalence of anti-Nc Abs but not quantitative characteristics of seropositivity.

**Figure 3:**
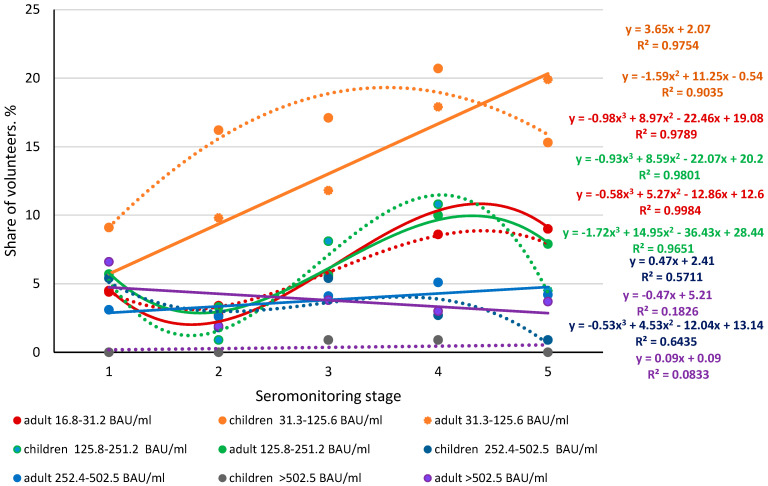
Distribution of anti-Nc antibody levels among children and adults, by seromonitoring stage. Colored lines—dynamics of Ab distribution in children and adults in the dynamics of monitoring; colored dotted lines—trends in Ab levels in children; solid color lines are trends in Ab levels in adults. The equations for the trend curves in their respective colors are shown to the right of the curves and correspond to the sequences in which the end points of the trend lines are located. See additional Table S2.

Analysis of the proportions of individuals with different levels of anti-Nc Abs showed two main trend line types. The distributions were quite close, both in form and levels, among children and adults in almost all stages of monitoring. The only exception was volunteers who had plasma Abs in the range of 252.4–502.5 BAU/ml (Figure 3). In this group, not only a predominance of Ab levels in stages 1–4 over adults was noted but also differences in trend curves. In children, the curve was described by a third-degree polynomial equation (y = −0.53x^3^ + 4.53 x^2^ − 12.04x + 13.14), while in adults it was described by a linear equation (y = 0.47x + 5.21). Determination coefficients in both cases exceeded 0.5. 

Interestingly, in all other groups, the Ab-level curves were close to each other. Among children in all the examined groups, a characteristic feature was noted: Ab levels increased to some extent from the first to the fourth stage; and they significantly decreased by the fifth stage. Moreover, the trend line for children was described by a third-degree polynomial curve of the type y = −ax^3^ + bx^2^ − cx + d. It seems that this is due to the previously described relatively short circulation time of anti-Nc Abs [35,36]. Interestingly, in volunteers with anti-Nc Abs levels >502.5 BAU/ml, the trend line was described by an equation of the form y = ax + b, possibly due to the small sample of volunteers with high anti-Nc Abs levels.

### 3.3. Seroprevalence of Anti-RBD Abs in the Volunteer Cohort, by Monitoring Stage

**Figure 4:**
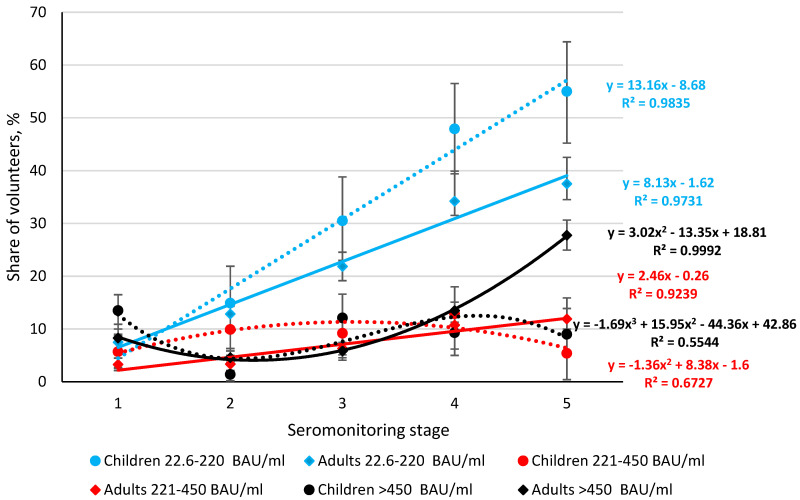
Distribution dynamics of anti-RBD antibody levels among children and adults, by seromonitoring stage. Colored lines are Ab distribution trends in children (dashed) and adults (solid). Trend equations and determination coefficients, in their corresponding colors, are shown on the right side of the graph next to their curves. Black vertical lines are 95% confidence intervals. Antibody values are given in BAU/ml (Table S3).

The dynamics of anti-RBD Ab seroprevalence were generally comparable to those of Nc (Figure 2). In stages 1 to 3, there were no differences in anti-RBD Ab seroprevalence in children and adults, although there was a tendency for higher seropositivity in children. In the fourth stage, this trend became statistically significant. In the fifth stage, in the context of a further increase in the share of seropositive persons among adults, there was a decreasing trend curve in children (Figure 4, Table S3).

The largest increase in the share of seropositive volunteers was noted in the group with minimal Ab levels (22.6–220 BAU/ml). This growth was comparable in children and adults. The growth trends (children, adults) were linear, described by the equations y = 13.16x − 8.68 and y = 8.13x − 1.62, respectively. At the same time, the process of increasing seropositivity in children was noticeably higher. This is evidenced by higher regression equation coefficients and statistically significant differences in area (from stage 3 to 5) along the *y*-axis (Figure 4).

The share of volunteers with Ab levels in the range 221–450 BAU/ml was significantly lower in all monitoring stages starting from stage 2 (*p* < 0.05). For adults, the trend was linear, described by a regression equation of the form y = 2.46x − 0.26. For children, it changed to a second-degree polynomial curve (y = −1.36x^2^ + 8.3829x − 1.6). The increase in the share of seropositive individuals occurred in the third monitoring stage, and the *y*-axis values at the start and end points were almost the same: 5.7% (95% CI: 2.5–10.9) versus 5.4% (95% CI: 2.0–11.4). 

In serogroup 3 (Abs > 450 BAU/ml), linear trends in children and adults transformed into polynomials, which could indicate greater heterogeneity within the group. In children, the degree of heterogeneity of the response increased to a polynomial of the third degree, described by the equation y = −1.69x^3^ + 15.95x^2^ − 44.36x + 42.86. In adults, the trend transformed into a second-degree polynomial curve: y = 3.02x^2^ − 13.35x + 18.81.

Thus, quantitative analysis of the structure of SARS-CoV-2 seroprevalence showed that the share of individuals with relatively low anti-RBD Ab levels increased during the progression of the COVID-19 epidemic: from 5.7% (95% CI: 2.5–10.9) in the first stage up to 55% (95% CI: 45.2–64.4) in the fifth. In adults, the growth was slightly less: 7.4% (95% CI: 6.0–9.0) in the first stage; and 39% (95% CI: 36.0–42.1) in the fifth stage (Table S3).

As the level of Abs to RBD increased, the heterogeneity of the response increased concomitantly with a decrease in the share of seropositive individuals in both children and adults. Trend lines shifted from linear to second- or third-degree polynomials. A possible reason may be a gradual decrease in the level of anti-RBD Abs in volunteers with overt or asymptomatic forms of COVID-19 within 6–8 months after the initial infection. In addition, the high frequency of asymptomatic forms, so characteristic of COVID-19, may also contribute to the development of a low humoral response. It is known, in particular, that Abs in asymptomatic, infected individuals can decrease to minimal values after two months [37,38].

### 3.4. Frequency of Asymptomatic COVID Forms, by Seromonitoring Stage 

**Figure 5:**
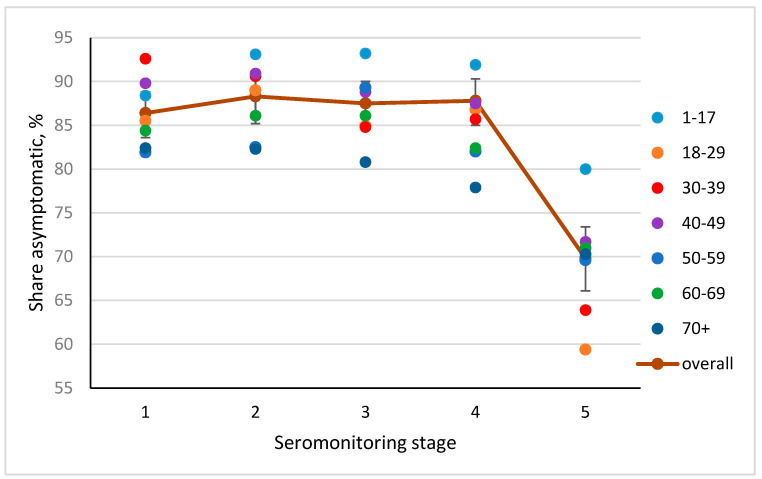
Share of asymptomatic forms among Nc–seropositive volunteers. The solid curve is the median share of seropositive volunteers with asymptomatic infection. Key: black vertical lines— [IQR]; colored dots—averaged values in each age group. Age groups of seropositive, asymptomatic volunteers are in years. See Table S4.

As noted, a characteristic feature of coronavirus infection is a significant proportion of patients with an asymptomatic course in which there are no clinical manifestations of disease yet Abs specific for SARS-CoV-2 Nc are produced. According to various sources, the number of such seropositive individuals can vary from 40% to 100% [14,16,38,39]. In the course of the staged survey of the St. Petersburg volunteer cohort, an absolute predominance of asymptomatic forms of infection (among SARS-CoV-2 seropositive individuals) was revealed (Figure 5, Table S4).

As follows from Figure 5 and Table S4, the share of people with asymptomatic forms of infection was relatively evenly distributed between age groups in all stages of seromonitoring. There were no statistically significant differences between age groups in stages 1–4. Significant differences were observed in stage 5 in the age groups 18–29 and 30–39 years old, 40–49 and 50–59 years old, and in the whole cohort (*p* > 0.05). At the same time, no differences were found in children aged 1–17 years. In the two older age groups, the decline was noticeable, although not statistically significant.

Such a decrease by the end of the second year of monitoring can probably be explained by the fact that the majority of volunteers had asymptomatic forms of infection in stages 1–4. Since seropositivity in asymptomatic infection is relatively short-lived [38], the proportion of such individuals by the fifth stage statistically significantly decreased.

The results presented describe the cohort as a whole. This is true for stages 1 to 3. However, by the beginning of 2021, a significant factor influencing the humoral immune response of the adult population was added to the situation: initiation of a vaccination campaign (Figure 1). By the fourth stage (ninth week of the year), 1.9% were vaccinated. This, however, had little effect on population seroprevalence in the fourth stage. The situation changed somewhat by the fifth stage, when at 35 weeks it increased to 26.3% (Figure 1). Although this vaccination level was about 40% of the threshold [6,7], it likely had a positive effect on SARS-CoV-2 Ab seroprevalence.

### 3.5. Post-Infectious and Post-Vaccination Ab Seroprevalence (Nc, RBD) in St. Petersburg Volunteers during Monitoring

The result of COVID-19 convalescence and/or SARS-CoV-2 vaccination is the formation of a specific response, manifested by cellular and humoral immune responses. Features of cellular immunity are considered in a number of fundamental reviews [40,41,42,43,44] and are not the subject of this work. Depending on the goals and objectives, various immune markers can be analyzed. For example, the three main immunoglobulin subtypes can be studied (IgM, IgG, IgA) in plasma, serum, or other body fluids [45,46]. Sometimes one subtype, most often IgG [47,48], is used, as in this work, wherein specific anti-Nc and anti-RBD IgG Ab contents were determined throughout seromonitoring. 

**Figure 6:**
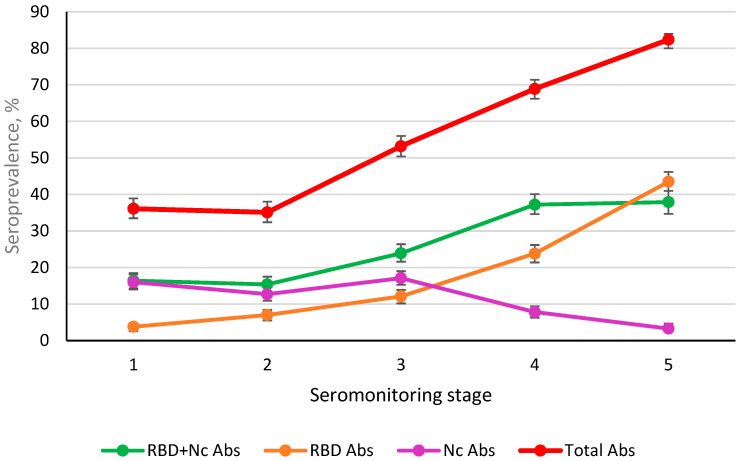
Seroprevalence of anti-Nc and anti-RBD IgG in volunteer plasma, by seromonitoring stage. Key: RBD + Nc double-positive volunteers with both Abs; RBD Abs—volunteers with only RBD Abs; Nc Abs—volunteers with only Nc Abs; Total Abs—total number of seropositive volunteers (RBD + Nc Abs, RBD Abs, Nc Abs) relative to all examined; vertical black lines—95% CI.

The obtained results showed that in the first stage, carried out at the sixth month, the SARS-CoV-2 specific seropositivity was mostly due to anti-Nc Abs (Figure 6). At the same time, the share of double-positive volunteers was equal to the share of volunteers who were monopositive for anti-Nc Abs. The share of individuals with only RBD Abs was 3.8% (95% CI: 2.8–5.0). By the second stage, the ratio remained practically unchanged, with the exception of an almost twofold increase in the share of individuals seropositive for RBD Abs (7.0% (95% CI: 5.6–8.5), *p* < 0.001). Stage 3 was marked by a significant increase in the share of double-positive volunteers (*p* < 0.001) and those seropositive for RBD only (*p* < 0.001). The total share of those mono and double positive for RBD Abs increased from 22.3% (95% CI: 20.1–24.8) to 36.1% (95% CI: 33.4–37.8); the differences were significant at *p* < 0.001. It was interesting to compare the increases in the shares of those monopositive for RBD and Nc Abs and those monopositive for Ab. In the former, the increase was 1.7-fold. In the latter, it was only 1.3-fold. Thus, already in the third stage, there was a slight increase in the share of Nc-seropositive people against the background of a significant increase in the share of those who were RBD seropositive (*p* < 0.05).

Starting from the fourth stage (Table 1), there was a significant increase in the total share of RBD monopositive individuals from 36.1% (95% CI: 33.4–37.8) to 61.1% (95% CI: 58.3–63.8), or growth by 1.7-fold (*p* < 0.0001). In parallel, the share of Nc-monopositive individuals decreased from 17.1% (95% CI: 15.0–19.3) to 7.8% (95% CI: 6.3–9.4), or 2.2-fold (*p* < 0.0001). Even more pronounced changes were observed in the fifth stage of seromonitoring conducted in August 2021 (Table 2). The share of RBD monopositive individuals increased from 19.5% (95% CI: 17.1–22.1) to 40.4% (95% CI: 37.3–43.5), *p* < 0.001. At the same time, there was no increase in double-positive volunteers (37.9%, 95% CI: 34.7–41.0), possibly due to a decrease in the level of anti-Nc Abs (Figure 6). The increase in the share of RBD carriers was significant. Hence, in the fourth stage, the increase in the RBD immunity level reached 11.4% (95% CI: 9.7–13.3). In the fifth stage, it was 39% (95% CI: 36.1–42.2). This is quite consistent with the level of vaccination, which in the 35th week of August 2021 was 26.3%. Considering that from the fourth stage the vaccination rate began to increase noticeably, it can be assumed that this process contributed to the increase in post-vaccination SARS-CoV-2 Ab seroprevalence (Table 1).

Serological testing revealed four groups of volunteers: double positive (RBD^+^ Nc^+^); positive for one Ag (RBD^+^ or Nc^+^); and individuals negative for both antigens (RBD^−^ Nc^−^) (Table 1). In the total cohort, the share of RBD^−^ Nc^−^ individuals was 21.2% (95% CI: 18.7–23.8). Among examined individuals, the data showed that about a third were RBD^+^ Nc^+^; about a fifth were RBD^+^ (only); and about 8% were Nc^+^ (only).

In the fourth stage, only 13.1% were vaccinated (95% CI: 11.1–15.3). In this group, the share of double positive remained practically unchanged, while the proportion of RBD^+^ increased by 2.5-fold, which is obviously a consequence of vaccination. Given the known general pattern of seropositivity, it was important to assess the contribution of vaccination to the overall pattern. As expected, the largest contribution to Ab seroprevalence in the cohort was probably due to vaccination. The total proportion of seropositive (RBD^+^ Nc^+^ and RBD^+^) was: 14.4% (95% CI: 12.3–16.7). In the period between the fourth and fifth stages, active vaccination of the population continued. Therefore, an increase in the proportion of volunteers seropositive for both antigens was be expected. As in the fourth stage, analysis of those vaccinated was carried out only among those who received Sputnik V.

The results of statistical processing for the total cohort are in good agreement with the previous analysis (Figure 6). The share of double-positive volunteers increased insignificantly (only 1.2-fold), while the share of those who were RBD^+^ increased 1.8-fold (*p* < 0.001). The share of Nc^+^ volunteers decreased by twofold (*p* < 0.001). The vaccination of 419 (41.9%) people did not significantly affect the proportion of double-positive volunteers, compared with stage 4 results (Table 1). This may indicate that within one volunteer group, vaccination mainly contributes to qualitative harmonization of the structure and, to a lesser extent, an increase in the quantitative indicator of RBD positivity (dual + RBD^+^). In the fourth stage, the latter already amounted to 87% (95% CI: 80.0–92.3). By the fifth stage, it had increased to 93.2% (95% CI: 90.5–95.5) (differences not statistically significant). Interestingly, in the context of a similar stabilization of the immune response to RBD, the proportion of Nc-monopositive individuals decreased by 5.4-fold. By stage 5, the proportion of individuals without these Abs decreased by 1.8% (Table 2).

As in the fourth stage, the actual contribution of the humoral response to SARS-CoV-2 was calculated. Overall seropositivity for all indicators (double^+^, RBD^+^, Nc^+^) for the entire cohort was 82.4% (95% CI: 79.9–84.7). Regarding the contribution to total Ab seroprevalence of persons vaccinated in stage 5, it was 39.4% (95% CI: 36.4–42.5).

In summarizing the results of the seroprevalence assessment in terms of Ab dynamics (Nc, RBD), we note that this process in the first three stages was due to the Nc response in individuals who underwent overt or asymptomatic infection. The situation began to change in the spring of 2021 with the start of SARS-CoV-2 vaccination, which changed the serological landscape. By the end of the second week of 2022, the level of those completely vaccinated was close to the 50% threshold [1]. Taking into account the increase in the level of post-vaccination immunity, we can assume that the population of St. Petersburg has reached, or will soon reach, the threshold level of population immunity. Thus, according to the official portal of the St. Petersburg administration, the value of Ro was 0.93 as of 8 January 2021 [49]. However, it is still too early to believe that victory over COVID-19 has been achieved in St. Petersburg. A serious threat that may nullify all efforts to achieve sustainable population immunity could be the rapid spread of the new omicron SARS-CoV-2 strain around the world [50,51,52].

## 4. Discussion

When assessing the overall COVID-19 situation in the city, it can be assumed that the pandemic near the end of the year (end of 2021 and the first ten days of January 2022) entered a phase of uneven stabilization in St. Petersburg. As of 9 January 2022, 2,732,661 people had completed double vaccination, representing 50.75% (95% CI: 50.71–50.79), while the distribution coefficient (similar to Ro) was 0.98 [49]. As a consequence of these data, starting from the 47th week of 2021, there has been a downward trend in the number of SARS-CoV-2 infections. Can this still unstable situation be considered proof of the end of the COVID-19 epidemic? Not yet, it seems. The generally accepted level of threshold immunity, 65% [4,5,6], has not been reached. Further, the Ro value is only slightly (0.3–0.2) below 1.0, barely below the level at which infection rates begin to decrease [5,6]. According to official data (as of 9 January 2022), the infection rate exceeds the recovery rate (1323 cases versus 1228 recovered) [49]. Such direct data indicate the need for further intensification of the vaccination campaign.

The structure of Ab seroprevalence in St. Petersburg has evolved over the 2 years of the COVID-19 epidemic. Initially during 2020, it was formed exclusively due to anti-Nc Abs, which is explained by the lack of vaccines and effective methods of prevention and therapy. The latter was mainly reduced to the relief of certain symptoms of acute infection [53,54,55]. In this regard, the humoral response remained the main method of control as a tool for regulating the infectious process [3,4,5,6,15,16,17]. Monitoring of SARS-CoV-2 seropositivity among the population in the first stages showed two unidirectional processes: simultaneous increases in the levels of Abs (to both Nc and RBD) in both children and adults (Figure 2). Starting from stage 4, these curves diverged: anti-Nc Ab levels decreased, while the share of individuals with anti-RBD Abs continued to increase rapidly. The latter reached 79.4% by stage 5 (95% CI: 76.6–82.0) in adults and 72.0% (95% CI: 62.7–80.2) in children.

Quantitative analysis of the immune response to Nc showed a dominance of a seropositive children and adults with Abs in the range of 37.4–150.0 BAU/ml. In adults, the trend was linear, described by the equation y = 3.65x + 2.07. In children, the dependence was more complex, with an increase in stages 2–4, and decreases in stages 1 and 5. The curve was described by the second-degree polynomial equation: y = 1.529x^2^ + 11.247x − 0.54. In all other groups, Ab levels were not statistically different from each other. At the same time, the general trend was an S-shaped curve for children in serogroups 1, 3, and 4, characterized by decreases in Ab level in stages 2 and 5. The trend line was described by a third-degree polynomial of the form: y = ax^3^ + bx^2^ − cx + d. As for the distribution curve of Ab levels among adults, the groups with minimum and average levels (20.0–37.2 and 150.2–300.0 BAU/ml) featured polynomial curves of the third degree. At higher levels (300.2–600.0 and >600 BAU/ml), linear dependences of the form y = ax + b were noted. In addition, a decrease in the share of Nc-seropositive individuals in most serogroups was most often observed.

Quantitative analysis of RBD found that, in most volunteers, content did not exceed 220 BAU/ml. The proportion of such individuals increased linearly throughout all stages of monitoring in both children and adults (Figure 4). Simultaneous with increasing levels of circulating anti-RBD Abs, an increase in dispersion was noted. This was manifested as a change in the linear trend to a polynomial. In adult volunteers with Ab levels in the 221–450 BAU/ml range, linearity was still preserved yet, in children, the curve was already described by a second-degree polynomial (Figure 4). A further increase in the anti-RBD IgG level transformed the trend line into a second-degree polynomial curve in adults and a third-degree polynomial curve in children. Thus, these results clearly show that the homogeneity of the Ab response depends on IgG level. In most individuals, blood Abs are contained at levels of 22.5–220 BAU/ml. With increases above 221 BAU/mL, the proportion of individuals with medium and high levels decreases, and the variance of the results increases, although not significantly (Figure 4).

One of the likely reasons for this phenomenon may be a relatively rapid decrease in Ab levels in seropositive individuals with asymptomatic forms of infection [39,56]. Analysis of these forms confirmed existing opinion about a significant prevalence among seropositive individuals. In stages 1 to 4 of monitoring, the proportion of such individuals stably varied within 81–93%. Then, in stage 5, it decreased in all age groups, except for children, by 60–70%. In trying to understand the reasons for this phenomenon, we assumed that, by this period, the majority of volunteers had encountered the virus to some degree. In most of them, the pathogen caused a short-term asymptomatic form of infection, accompanied by a short circulation of specific Abs. By the fifth stage, the number of such individuals decreased markedly. This was likely reflected as a decrease in the share of seropositive persons among those with asymptomatic infection.

As noted, monitoring was carried out for two years, i.e., throughout the COVID-19 epidemic. One of the important tasks of this monitoring was to determine the dynamics of volunteer seroconversion (anti-Nc, anti-RBD). Analysis of these Abs in the first three stages showed a predominance of Nc seropositivity with a minimal proportion of individuals seropositive for RBD (Figure 6). From the third to the fifth stages, two opposite and simultaneous processes were noted: a decrease in Nc Abs level to 3.3% (95% CI: 2.4–4.4) and an increase in the share of RBD seropositive to 40.4% (95% CI: 37.4–43.5). At the same time, starting from the fourth stage, a new factor appeared in the structure of seropositivity: post-vaccination seropositivity for RBD due to immunization of St. Petersburg residents with the Sputnik V (GamCOVIDVac) vaccine, which accounted for about 80% of all vaccinations. In this regard, it seemed important to assess the contribution of post-vaccination immunity to overall volunteer seroprevalence.

The overall seropositivity level in stage 4 was 68.7% (95% CI: 65.8–71.6). Among those vaccinated with the Sputnik V(GamCOVIDVac) vaccine, it was 90.8% (95% CI: 84.6–95.2), of which the proportion of RBD-positive persons (RBD^+^ Nc^+^ + RBD^+^) was 87.0% (95% CI: 80.0–92.2). If we relate the proportion of these individuals to a total cohort of 1000 people, then their contribution to overall seropositivity is 11.5% (95% CI: 9.6–13.7).

As far as the analogous calculation for stage 5: overall seropositivity was 82.4% (95% CI: 79.9–84.7). The share of RBD-positive persons (RBD^+^ Nc^+^ + RBD^+^) among 419 vaccinated was 93.3% (95% CI 90.5–95.5). When this proportion is related to the cohort as a whole (1000 people), the contribution of RBD^+^ vaccinated individuals would be 39.1% (95% CI: 36.1–42.2). Thus, an increase in the share vaccinated from 1.9% (stage 4) to 26.3% (stage 5) was able to confer an increase in the contribution of post-vaccination immunity to cohort seroprevalence from 11.5% to 39.1%, or 3.4-fold.

The results presented testify in favor of the important role of vaccination in the formation of herd immunity. In this regard, it is impossible not to agree with the opinion of researchers [49,50] that vaccination against SARS-CoV-2 has proven to be effective, with essentially no alternatives, in overcoming the COVID-19 pandemic.

## Figures and Tables

**Table 1:** Structure of antibody seroprevalence in the fourth stage.

	Total Cohort (*n* = 1000)		Vaccinated (*n* = 131)	
	Nc^+^, *n* % (95% CI)	Nc^−^*, n* % (95% CI)	Nc^+^, *n* % (95% CI)	Nc^−^, *n* % (95% CI)
**RBD+, *n***	381	223	41	73
**% (95% CI)**	38.1 (35.1–41.3)	22.3 (19.8–25.1)	31.3 (23.5–40)	55.7 (46.8–64.4)
**RBD−, *n***	82	212	5	12
**% (95% CI)**	8.2 (6.6–10.1)	21.2 (28.4–34.2)	3.8 (1.2–8.7)	9.2 (4.8–15.0)

Note: RBD^+^—anti-RBD Abs detected; Nc^+^—anti-Nc Abs detected; RBD^−^ and Nc^−^—the corresponding Abs not detected. In each cell: *n*—number; percentage of surveyed; and 95% confidence interval.

**Table 2:** Structure of antibody seroprevalence in the fifth stage.

	Total Cohort (*n* = 1000)		Vaccinated (*n* = 419)	
	Nc^+^, *n* % (95% CI)	Nc^−^, *n* % (95% CI)	Nc^+^, *n* % (95% CI)	Nc^−^, *n* % (95% CI)
**RBD+, *n***	379	404	132	259
**% (95% CI)**	37.9 (34.9–41.0)	40.4 (37.3–43.5)	36.3 (31.7–41.1)	61.8 (56.9–66.40)
**RBD−, *n***	41	176	361.8	25
**% (95% CI)**	4.1 (3.0–5.5)	17.6 (15.3–20.1)	(57.0–66.5)	6.0 (3.9–8.6)

Notes: same as Table 1.

## Data Availability

The authors confirm that the data supporting the findings of this study are available within the article and/or its Supplementary Materials.

## Supplementary Materials

The following supporting information can be downloaded at: https://www.mdpi.com/article/10.3390/v14050913/s1, Table S1: Dynamics of incidence and vaccination of the population of St. Petersburg in 2021; Table S2: Distribution of proportions of seropositivity to N and RBD ags in children and adult volunteers of the surveyed age groups at the stages of seromonitoring; Table S3: Dynamics of incidence and vaccination of the population of St. Petersburg in 2021; Table S4: The proportion of asymptomatic forms of COVID-19 among seropositive volunteers at the stages of seromonitoring.

## References

[1] Coronavirus-Monitor. https://coronavirus-monitor.info/.

[2] https://www.gov.spb.ru/press/governor/225782/.

[3] Yadegari I., Omidi M., Smith S.R. (2021). The herd-immunity threshold must be updated for multi-vaccine strategies and multiple variants. Sci. Rep..

[4] Xia Y., Zhong L., Tan J., Zhang Z., Lyu J., Chen Y., Zhao A., Huang L., Long Z., Liu N.-N. (2020). How to Understand “Herd Immunity” in COVID-19 Pandemic. Front. Cell Dev. Biol..

[5] Fontanet A., Cauchemez S. (2020). COVID-19 herd immunity: Where are we?. Nat. Rev. Immunol..

[6] Randolph H.E., Barreiro L.B. (2020). Herd Immunity: Understanding COVID-19. Immunity.

[7] Liu Y., Gayle A.A., Wilder-Smith A., Rocklöv J. (2020). The reproductive number of COVID-19 is higher compared to SARS coronavirus. J. Travel. Med..

[8] Jin Y., Yang H., Ji W., Wu W., Chen S., Zhang W., Duan G. (2020). Virology, Epidemiology, Pathogenesis, and Control of COVID-19. Viruses.

[9] Sanche S., Lin Y.T., Xu C., Romero-Severson E., Hengartner N., Ke R. (2020). High Contagiousness and Rapid Spread of Severe Acute Respiratory Syndrome Coronavirus 2. Emerg. Infect. Dis..

[10] Jefferson T., Del Mar C.B., Dooley L., Ferroni E., Al-Ansary L.A., Bawazeer G.A., van Driel M.L., Jones M.A., Thorning S., Beller E.M. (2020). Physical interventions to interrupt or reduce the spread of respiratory viruses. Cochrane Database Syst. Rev..

[11] Al-Betar M.A., Alyasseri Z.A.A., Awadallah M.A., Doush I.A. (2020). Coronavirus herd immunity optimizer (CHIO). Neural Comput. Appl..

[12] Dopico X.C., Ols S., Loré K., Hedestam G.B.K. (2021). Immunity to SARS-CoV-2 induced by infection or vaccination. J. Intern. Med..

[13] Díaz-Salazar C., Sánchez-García A., Rodríguez-Gutiérrez R., Camacho-Ortiz A., Saldívar-Rodríguez D., González-González J.G. (2021). Prevalence and associated characteristics of anti-SARS-CoV-2 antibodies in Mexico 5 months after pandemic arrival. BMC Infect. Dis..

[14] Popova A.Y., Ezhlova E.B., Mel’nikova A.A., Bashketova N.S., Fridman R.K., Lyalina L.V., Smirnov V.S., Chkhindzheriya I.G., Grechaninova T.A., Agapov K.A. (2020). Herd Immunity to SARS-CoV-2 among the Population in Saint-Petersburg during the COVID-19 Epidemic. Probl. Osob. Opasnykh Infektsii = Probl. Part. Danger. Infect..

[15] Popova A.Y., Andreeva E.E., Babura E.A., Balakhonov S.V., Bashketova N.S., Bulanov M.V., Valeullina N.N., Goryaev D.V., Detkovskaya N.N., Ezhlova E.B. (2021). Features of developing SARS-CoV-2 nucleocapsid protein population-based seroprevalence during the first wave of the COVID-19 epidemic in the Russian Federation. Russ. J. Infect. Immun. = Infektsiya Immun..

[16] Popova A.Y., Smirnov V.S., Andreeva E.E., Babura E.A., Balakhonov S.V., Bashketova N.S., Bugorkova S.A., Bulanov M.V., Valeullina N.N., Vetrov V.V. (2021). SARS-CoV-2 seroprevalence structure of the russian population during the COVID-19 pandemic. Viruses.

[17] Popova A.Y., Ezhlova E.B., Melnikova A.A., Smirnov V.S., Lyalina L.V., Kozlovskikh D.N., Luchinina S.V., Romanov S.V., Valeullina N.N., Dikonskaya O.V. (2021). Peculiarities of seroprovalence to SARS-CoV-2 in the population of the Middle and Southern Urals in the early period of the COVID-19 pandemic. Epidemiol. Vaccinal Prev..

[18] Popova A.Y., Smirnov V.S., Ezhlova E.B., Melnikova A.A., Trotsenko O.E., Kurganova O.P., Detkovskaya T.N., Lyalina L.V., Abbasova E.I., Yurgina O.M. (2021). Herd immunity to SARS-CoV-2 in the population of the Southern regions of the Far East of Russia. Acta Biomed. Sci..

[19] Popova A.Y., Andreeva E.E., Babura E.A., Balakhonov S.V., Bashketova N.S., Bugorkova S.A., Bulanov M.V., Valeullina N.N., Goryaev D.V., Detkovskaya N.N. (2021). Peculiarities of SARS-CoV-2 nucleocapsid in children during the COVID-19 epidemic of 2020. Pediatriya Named After GN Speransky.

[20] Popova A.Y., Ezhlova E.B., Melnikova A.A., Smirnov V.S., Lyalina L.V., Goryaev D.V., Khodov D.A., Chepizhko T.G., Rusin M.V., Kuznetsova N.N. (2021). Structure of herd immunity to SARS-CoV-2 in the Krasnoyarsk region population in the COVID-19 epidemic. Acta Biomed. Sci..

[21] Popova A.Y., Tarasenko A.A., Smolenski V.Y., Egorova S.A., Smirnov V.S., Dashkevich A.M., Svetogor T.N., Glinskaya I.N., Skuranovich A.L., Milichkina A.M. (2021). Herd immunity to SARS-CoV-2 among the population of the Republic of Belarus amid the COVID-19 pandemic. Russ. J. Infect. Immun. = Infektsiya Immun..

[22] Popova A.Y., Kasymov O.T., Smolenski V.Y., Smirnov V.S., Egorova S.A., Nurmatov Z.S., Milichkina A.M., Suranbaeva G.S., Khamitova I.V., Zueva E.V. (2021). SARS-CoV-2 Herd Immunity of the Kyrgyz Population in 2021. Res. Sq..

[23] Popova A.Y., Totolian A.A. (2021). Methodology for assessing herd immunity to the SARS-CoV-2 virus in the context of the COVID-19 pandemic. Russ. J. Infect. Immun. = Infektsiya Immun..

[24] WHO Protocol for a Population-Based Age-Stratified Sero-Epidemiological Study of COVID-19 Infection in Humans. Version 2.0. 2020. https://apps.who.int/iris/bitstream/handle/10665/332188/WHO-2019CoV-Seroepidemiology-2020.2-rus.pdf.

[25] Logunov D.Y., Dolzhikova I.V., Zubkova O.V., Tukhvatulin A.I., Shcheblyakov D.V., Dzharullaeva A.S., Grousova D.M., Erokhova A.S., Kovyrshina A.V., Botikov A.G. (2020). Safety and immunogenicity of an rAd26 and rAd5 vector-based heterologous prime-boost COVID-19 vaccine in two formulations: Two open, non-randomised phase 1/2 studies from Russia. Lancet.

[26] Jonesa I., Royb P. (2021). Sputnik V COVID-19 vaccine candidate appears safe and effective. Lancet.

[27] Ryzhikov A.B., Ryzhikov E.A., Bogryantseva M.P., Usova S.V., Danilenko E.D., Nechaeva E.A., Pyankov O.V., Pyankova O.G., Gudymo A.S., Bodnev S.A. (2021). A single blind, placebo-controlled randomized study of the safety, reactogenicity and immunogenicity of the “EpiVacCorona” Vaccine for the prevention of COVID-19, in volunteers aged 18–60 years (phase I–II). Russ. J. Infect. Immun. = Infektsiya Immun..

[28] Wald A., Wolfowitz J. (1939). Confidence Limits for Continuous Distribution Functions. Ann. Math. Stat..

[29] Agresti A., Coull B.A. (1998). Approximate Is Better than “Exact” for Interval Estimation of Binomial Proportions. Am. Stat..

[30] Significant Difference Calculator (z-Test) RADAR Research Company. https://radar-research.ru/software/z-test_calculator.

[31] Del Rio C., Omer S.B., Malani P.N. (2021). Winter of Omicron—The Evolving COVID-19 Pandemic. JAMA.

[32] Mohiuddin M., Kasahara K. (2021). Investigating the aggressiveness of the COVID-19 Omicron variant and suggestions for possible treatment options. Respir. Med..

[33] Mahase E. (2021). COVID-19: Omicron and the need for boosters. BMJ.

[34] Yang H.S., Costa V., Racine-Brzostek S.E., Acker K.P., Yee J., Chen Z., Karbaschi M., Zuk R., Rand S., Sukhu A. (2021). Association of Age With SARS-CoV-2 Antibody Response. JAMA Netw. Open.

[35] Rosati M., Terpos E., Ntanasis-Stathopoulos I., Agarwal M., Bear J., Burns R., Hu X., Korompoki E., Donohue D., Venzon D.J. (2021). Sequential Analysis of Binding and Neutralizing Antibody in COVID-19 Convalescent Patients at 14 Months After SARS-CoV-2 Infection. Front. Immunol..

[36] Koerber N., Priller A., Yazici S., Cheng C.-C., Mijočević H., Wintersteller H., Jeske S., Vogel E., Feuerherd M., Tinnefeld K. (2022). Dynamics of spike-and nucleocapsid specific immunity during long-term follow-up and vaccination of SARS-CoV-2 convalescents. Nat. Commun..

[37] Wu J., Liang B.-Y., Fang Y.-H., Wang H., Yang X.-L., Shen S., Chen L.-K., Li S.-M., Lu S.-H., Xiang T.-D. (2021). Occurrence of COVID-19 Symptoms During SARS-CoV-2 Infection Defines Waning of Humoral Immunity. Front. Immunol..

[38] Sayampanathan A.A., Heng C.S., Pin P.H., Pang J., Leong T.Y., Leea V.J. (2021). Infectivity of asymptomatic versus symptomatic COVID-19. Lancet.

[39] Almadhi M.A., Abdulrahman A., Sharaf S.A., AlSaad D., Stevenson N.J., Atkin S.L., AlQahtani M.M. (2021). The high prevalence of asymptomatic SARS-CoV-2 infection reveals the silent spread of COVID-19. Int. J. Infect. Dis..

[40] Jarjour N.N., Masopust D., Jameson S.C. (2021). T Cell Memory: Understanding COVID-19. Immunity.

[41] De Candia P., Prattichizzo F., Garavelli S., Matarese G. (2021). T Cells: Warriors of SARS-CoV-2 Infection. Trends Immunol..

[42] Grifoni A., Sidney J., Vita R., Peters B., Crotty S., Weiskopf D., Sette A. (2021). SARS-CoV-2 human T cell epitopes: Adaptive immune response against COVID-19. Cell Host Microbe.

[43] Sette A., Crotty S. (2021). Adaptive immunity to SARS-CoV-2 and COVID-19. Cell.

[44] Niessl J., Sekine T., Buggert M. (2021). T cell immunity to SARS-CoV-2. Semin. Immunol..

[45] Alkharaan H., Bayati S., Hellström C., Aleman S., Olsson A., Lindahl K., Bogdanovic G., Healy K., Tsilingaridis G., De Palma P. (2021). Persisting Salivary IgG Against SARS-CoV-2 at 9 Months After Mild COVID-19: A Complementary Approach to Population Surveys. J. Infect. Dis..

[46] Huynh A., Arnold D.M., Smith J.W., Moore J.C., Zhang A., Chagla Z., Harvey B.J., Stacey H.D., Ang J.C., Clare R. (2021). Characteristics of Anti-SARS-CoV-2 Antibodies in Recovered COVID-19 Subjects. Viruses.

[47] Mahallawi W., Alzahrani M., Alahmadey Z. (2021). Durability of the humoral immune response in recovered COVID-19 patients. Saudi J. Biol. Sci..

[48] Gundlapalli A.V., Salerno R.M., Brooks J.T., Averhoff F., Petersen L.R., McDonald L., Iademarco M.F. (2020). CDC COVID-19 Response. ARS-CoV-2 Serologic Assay Needs for the Next Phase of the US COVID-19 Pandemic Response Open. Forum Infect. Dis..

[49] The Coronavirus in SPB. https://www.gov.spb.ru/covid-19/.

[50] Thakur V., Ratho R.K. (2021). OMICRON (B.1.1.529): A new SARS-CoV-2 variant of concern mounting worldwide fear. J. Med. Virol..

[51] Kannan S.R., Spratt A.N., Sharma K., Chand H.S., Byrareddy S.N., Singh K. (2022). Omicron SARS-CoV-2 variant: Unique features and their impact on pre-existing antibodies. J. Autoimmun..

[52] Russell R.S. (2021). Omicron: A Speculation on Its Potential Superpowers. Viral Immunol..

[53] Majumder J., Minko T. (2021). Recent Developments on Therapeutic and Diagnostic Approaches for COVID-19. AAPS J..

[54] Gavriatopoulou M., Ntanasis-Stathopoulos I., Korompoki E., Fotiou D., Migkou M., Tzanninis I.-G., Psaltopoulou T., Kastritis E., Terpos E., Dimopoulos M.A. (2021). Emerging treatment strategies for COVID-19 infection. Clin. Exp. Med..

[55] Smirnov V.S., Totolian Areg A. (2020). Some opportunities for immunotherapy in coronavirus infection. Russ. J. Infect. Immun..

[56] Boyton R.J., Altmann D.M. (2021). The immunology of asymptomatic SARS-CoV-2 infection: What are the key questions. Nat. Rev. Immunol..

