# Supplementary material for: Longitudinal Randomized Cohort Study of SARS-CoV-2 Antibody Seroprevalence in the St. Petersburg Population
Source: Viruses. 2022 Apr 27;14(5):913. doi: 10.3390/v14050913 (PMC9146723; doi:10.3390/v14050913)
Supplement: Supplementary file 1 [file viruses-14-00913-s001.zip › viruses-1651956-supplementary.pdf]

Table S1. Dynamics of incidence and vaccination of the population of St. Petersburg in 2021.

Week number 2021	Number of infected ‱o	Number of people vaccinated %
1	208.49	0
2	421.31	0
3	398.23	0
4	410.75	0
5	269.73	0.23
6	198.71	0.41
7	153.93	0.65
8	140.17	1.16
9	121.22	1.88
10	125.44	2.81
11	123.58	3.73
12	118.77	4.39
13	102.52	5.05
14	92.43	5.5
15	91.1	5.88
16	91.71	6.43
17	89.85	7.27
18	93.55	8.15
19	93.16	8.88
20	96.04	9.63
21	104.01	10.26
22	109.3	10.65
23	108.83	10.78
24	111.49	11.55
25	123.04	12.16
26	150.16	12.87
27	204.52	13.45
28	250.28	14.12
29	254.57	15.07
30	251.93	16.61
31	249.56	18.58
32	248.15	20.53

33	234.23	23.06
34	196.4	24.93
35	150.36	26.34
36	148.43	27.65
37	164.48	28.8
38	190.72	29.77
39	208.98	30.63
40	265.68	31.48
41	310.85	32.27
42	351.89	33.16
43	422.73	34.33
44	411.45	35.32
45	406.88	36.01
46	395.35	37.42
47	360.01	39.01
48	220.18	40.85
49	313.56	42.93
50	286.7	45.27
51	222.59	47.05

Table S2. Distribution of proportions of seropositivity to N and RBD ags in children and adult volunteers of the surveyed age groups at the stages of seromonitoring.

Age. years	Stages of the seromonitoring									
	1		2		3		4		5	
	Nc ab. % (95% CI)	RBD ab.% (95% CI)	Nc ab. % (95% CI)	RBD ab. % (95% CI)	Nc ab. % (95% CI)	RBD ab. % (95% CI)	Nc ab. % (95% CI)	RBD ab. % (95% CI)	Nc ab. % (95% CI)	RBD ab. % (95% CI)
1-17	37.0 (30.6-43.8)	22.7 (16.1-30.5)	47.2 (39.7-54.8)	26.2 (19.2-34.3)	54.6 (47.7-61.4)	51.8 (43.2-60.3)	46.5 (39.1-54.0)	70.0 (61.7-77.4)	30.9 (23.8-38.6)	74.7 (67.3-81.2)
18-29	24.1 (18.8-30)	16.7 (11.1-23.6)	29.2 (22.9-36.0)	18.0 (12.2-25.1)	38.4 (32.3-44.9)	32.0 (24.6-40.1)	38.6 (31.8-45.8)	60.0 (51.7-67.9)	37.9 (30.5-45.6)	83.4 (76.9-88.7)
30-39	27.5 (22.5-32.9)	16.6 (11.7-22.5)	25.0 (19.6-31.0)	19.4 (14.2-25.6)	34.8 (29.4-40.4)	30.3 (24.0-37.2)	45.6 (39.4-51.8)	57.8 (50.6-64.7)	36.0 (26.9-42.4)	77.0 (71.1-82.2)
40-49	23.6 (18.8-29)	17.2 (12.2-23.0)	24.3 (19.1-30.1)	20.6 (15.3-26.8)	34.0 (28.5-39.8)	37.7 (31.1-44.8)	41.9 (36.0-48.1)	60.8 (53.7-67.5)	42.2 (36.1-48.6)	80.5 (75-85.2)
50-59	30.2 (25.1-35.8)	22.5 (17.2-28.5)	27.9 (22.5-33.7)	24.7 (19.2-30.8)	40.2 (34.6-46.0)	34.8 (28.6-41.4)	43.3 (37.6-49.2)	57.1 (50.4-63.6)	39.4 (33.4-45.6)	81.5 (76.2-86.0)
60-69	33.0 (27.8-39.8)	22.1 (16.9-28.1)	29.3 (23.9-35.3)	24.8 (19.2-30.9)	40.7 (35.1-46.5)	34.5 (28.3-41.1)	46.7 (41.0-52.5)	63.3 (56.6-69.6)	54.1 (47.9-60.2)	82.5 (77.4-86.8)
70+	37.1 (29.1-45.5)	21.8 (14.2-31.3)	23.2 (16.1-31.6)	22.8 (15-32.2)	32.9 (25.2-41.2)	34.6 (25.5-44.8)	50.4 (41.6-59.1)	61.4 (51.2-70.9)	52.0 (42.8-61.1)	76.4 (67.9-83.6)
Overal l	29.7 (24.6-31.9)	20.1 (17.9-22.4)	29.1 (26.6-31.5)	22.4 (20.1-24.8)	39.3 (34.7-41.6)	36.1 (33.4-38.8)	44.5 (42.0-46.9)	61.0 8(58.3-63.8)	41.9 (39.4-44.5)	79.9 (77.7-1.9)

Table S3: Distribution of Abs to RBD ag levels among children and adults in the dynamics of seromonitoring.

Ages interval. years	Proportion of individuals with different levels of abs at different stages. % (95% CI)														
	1 stage; antibodies level. BAU/ml			2 srage; antibodies level. BAU/ml			3 stage; antibodies level. BAU/ml			4 stage; antibodies abs level. BAU/ml			5 stage; antibodies level. BAU/ml		
	22.6-220	221-450	>450	22.6-220	221-450	>450	22.6-220	221-450	>450	22.6-220	221-450	>450	22.6-220	221-450	>450
1-17	5.7 (2.5-10.9)	5.7 (2.5-10.9)	13.5 (8.3-17.2)	14.9 (9.5-21.9)	9.9 (5.5-16.1)	1.4 (0.2-5.0)	30.5 (23-38.8)	9.2 (5-15.2)	12.1 (7.2-18.6)	47.9 (39.4-56.5)	12.9 (7.8-19.6)	9.3 (5-15.4)	55.0 (45.2-64.4)	5.4 (2-11.4)	9.0 (4.4-15.9)
18-29	10.7 (6.2-16.7)	2.7 (0.7-6.7)	3.3 (1.1-7.6)	13.3 (8.3-19.8)	2.3 (0.7-7.0)	2.0 (0.4-5.7)	22 (15.6-29.5)	6.0 (2.8-11.1)	4.0 (1.5-8.5)	42.0 (34-50.3)	10.0 (5.7-15.9)	8.1 (4.2-13.6)	44.2 (34.9-53.9)	15.9 (9.7-24)	24.8 (17.1-33.8)
30-39	9.5 (5.8-14.5)	3.5 (1.4-7.1)	3.5 (1.4-7.1)	14.4 (9.6-20.1)	2.0 (0.5-5.0)	3.0 (1.1-6.4)	21.7 (16.2-28.1)	5.1 (2.1-9.1)	3.5 (1.4-7.1)	43.7 (36.7-50.9)	7.5 (4.3-12.1)	6.5 (3.5-10.9)	42 (34.2-50.2)	9.6 (5.4-15.3)	25.5 (18.9-33.0)
40-49	4.9 (2.4-8.8)	4.9 (2.4-8.8)	7.4 (4.2-11.8)	9.8 (6.1-14.7)	6.4 (3.4-10.6)	4.4 (2.0-8.2)	26 (20.1-32.6)	5.4 (2.7-9.4)	6.4 (3.4-10.6)	34.8 (28.3-41.8)	11.8 (7.7-17)	14.2 (9.7-19.8)	30.2 (23.5-37.7)	11.6 (7.2-17.4)	36.6 (29.4-44.0)
50-59	8.8 (5.5-13.3)	4.4 (1.1-8.0)	9.2 (5.8-13.8)	15.0 (10.6-20.3)	4.8 (2.4-8.5)	4.8 (2.4-8.5)	22.9 (17.6-28.9)	6.6 (3.8-10.7)	5.3 (2.7-9.1)	33.6 (27.5-40.2)	10.6 (6.9-15.4)	12.8 (8.8-17.9)	39.9 (32.7-47.4)	12 (7.7-17.6)	29.5 (23-36.7)
60-69	6.2 (3.4-10.2)	3.1 (1.2-6.3)	12.8 (8.8-17.9)	12.4 (8.4-17.4)	2.7 (1.0-5.7)	9.7 (6.2-14.4)	15.9 (11.4-21.4)	8.0 (4.8-12.3)	10.6 (6.9-15.4)	31.0 (25-37.4)	12.4 (8.4-17.4)	19.9 (14.9-25.7)	32.6 (26-39.8)	11.8 (7.5-17.3)	34.8 (28-42.1)
70+	5.0 (1.6-11.1)	3.0 (0.6-8.4)	13.9 (7.8-22.2)	9.9 (4.8-17.5)	4.0 (1.1-9.8)	8.9 (4.2-16.2)	14.9 (8.6-23.3)	9.9 (4.8-17.5)	9.9 (4.8-17.5)	28.7 (20.1-38.6)	10.9 (5.6-18.6)	21.7 (14.2-31.3)	35.1 (24.5-46.8)	13.0 (6.4-22.6)	26.0 (16.6-37.2)
Overall	7.4 (6.0-9.0)	3.9 (2.8-5.0)	8.8 (7.3-10.5)	13 (11.1-15)	4.5 (3.4-5.8)	5.0 (3.8-6.3)	22.1 (19.8-24.5)	6.9 (5.5-8.4)	7.1 (5.8-8.7)	37.2 (34.5-39.9)	10.8 (9.2-12.7)	13.1 (11.3-15.1)	39.0 (36-42.1)	11.3 (9.4-13.4)	28.0 (25.2-30.9)

Table S4: The proportion of asymptomatic forms of COVID-19 among seropositive volunteers at the stages of seromonitoring

Ages intervals years	Stages of seromonitoring				
	1. %(95% CI)	2. %(95% CI)	3. %(95% CI)	4. %(95% CI)	5. %(95% CI)
1-17	88.4(81.3-93.5)	93.1(86.2-97.2)	93.2(87.1-97.0)	91.9(83.9-97.0)	80(66.3-90.0)
18-29	85.5(76.1-92.3)	89.0(79.5-95.2)	84.9(76.0-91.5)	86.8(77.1-93.5)	59.4(46.3-71.5)#
30-39	92.6(85.4-97)	90.6(80.7-96.5)	84.8(76.4-91.0)	85.7(78.1-91.4)	63.9(52.9-74.0)#
40-49	89.8(81.5-95.2)	90.9(81.3-96.6)	88.8(80.8-94.3)	87.5(80.0-93.0)	71.7(62.1-80.0)
50-59	81.9(73.2-88.7)	82.5(72.4-90.1)	89.3(82.3-94.1)	82.0(74.1-88.1)	69.6(59.7-78.3)#
60-69	84.4(76.9-90.2)	86.1(76.9-92.6)	86.1(78.6-91.7)	82.4(75.1-88.3)	71(62.9-78.3)
70+	82.4(72.6-90.0)	82.3(65.5-93.2)	80.8(66.7-90.8)	77.9(66.2-87.1)	70.3(57.6-81.1)
Overall. Me [IQR]	86.4(83.6-88.8)	88.3(85.2-91.0)	87.5(84.8-90.0)	84.8(82.0-87.3)	69.8(66.1-73.4)*

Note: * Differences are statistically significant compared to the final data at other stages of monitoring.

Differences are statistically significant compared to data's at other stages monitoring of same ages interval.

Overall expressed as Me[IQR].

IQR - interquartile range.
